# Interaction of streptococcal plasminogen binding proteins with the host fibrinolytic system

**DOI:** 10.3389/fcimb.2013.00085

**Published:** 2013-11-22

**Authors:** Marcus Fulde, Michael Steinert, Simone Bergmann

**Affiliations:** ^1^Institute for Medical Microbiology and Hospital Epidemiology, Hannover Medical SchoolHannover, Germany; ^2^Department of Infection Biology, Institute of Microbiology, Technische Universität BraunschweigBraunschweig, Germany

**Keywords:** plasminogen, *Streptococcus*, enolase, M-protein, SCM, transmigration, phagocytosis

## Abstract

The ability to take advantage of plasminogen and its activated form plasmin is a common mechanism used by commensal as well as pathogenic bacteria in interaction with their respective host. Hence, a huge variety of plasminogen binding proteins and activation mechanisms exist. This review solely focuses on the genus *Streptococcus* and, in particular, on the so-called non-activating plasminogen binding proteins. Based on structural and functional differences, as well as on their mode of surface linkaging, three groups can be assigned: M-(like) proteins, surface displayed cytoplasmatic proteins with enzymatic activities (“moonlighting proteins”) and other surface proteins. Here, the plasminogen binding sites and the interaction mechanisms are compared. Recent findings on the functional consequences of these interactions on tissue degradation and immune evasion are summarized.

## Introduction

In addition to a largely commensal lifestyle reflected by colonization of mucosal surfaces, species like *S. pyogenes* (group A streptococci, GAS) and *S. pneumoniae* appear as opportunistic pathogens causing a wide spectrum of local and systemic diseases in humans. In agriculture, subclinical infections with *Streptococcus* sp. lead to high financial deficits and an increasing demand for pets fosters the occurrence of streptococcal species in companion animals (Devriese et al., 1986; Staats et al., 1997; van der Linden et al., 2009). Interspecies jumps which might result in real zoonoses, therefore, represent a new attribute of streptococcal infections (Tang et al., 2006; Chen et al., 2007; Fittipaldi et al., 2012; Fulde and Valentin-Weigand, 2013).

Despite the large number of streptococcal species present in a broad variety of different hosts, pathogenesis and clinical signs appears highly similar. This implies that comparable virulence traits exist. The utilization of plasminogen for bacterial adherence to cell surfaces, dissemination in the body, and protection against immune defense represents a complex pathomechanism that reflects the sophisticated adaptation of streptococci to their host environments. Although genetically closely related, streptococci harbor a broad variety of different plasminogen binding- and activation mechanisms. Therefore, this genus represents a suitable model for discussing bacteria-plasminogen interactions.

Plasminogen is a 92 kDa zymogen of the broad-spectrum serine protease plasmin. It is composed of an 8 kDa pre-activation peptide, five homologous amino acid stretches forming a triple loop structure (Kringle domains, K1–5), and a C-terminal protease domain (25 kDa). A functional attribute of the Kringle domains is their affinity to lysine (lysine-binding sites) in a very pronounced hierarchical order: K1>K3>K4>K5 except K2 which has negligible affinity to lysine (Miyashita et al., 1988; Marti et al., 1997). The protein comprising the first four Kringle domains (K1–4) shows inhibitory effect on angiogenesis and is, therefore, designated angiostatin. In contrast, the protein fragment containing the subdomain K5 and the serine protease domain is known as mini-plasminogen (mPLG). The detailed role of this fragment in physiology is still unknown.

In the fibrinolytic process, plasminogen is cleaved into the two-chained serine protease plasmin by two host-derived activators, the tissue-type plasminogen activator (tPA) and the urokinase-type plasminogen activator (uPA) (Dano et al., 1985). Plasmin-mediated cleavage of circulating native plasminogen (named Glu-plasminogen according to the first N-terminal amino acid) results in a truncated form of the zymogen called Lys plasminogen (~83 kDa), which is more rapidly converted into plasmin. Streptococci activate plasminogen either via an endogenously produced streptokinase (Castellino and Violand, 1979) or by subversion of the host-derived activators, tPA, and uPA. Irrespective of expression of a bacterial plasminogen activator, the additional subversion of host-derived activators clearly demonstrates that bacteria have evolved efficient compensatory strategies to gain benefit from recruited plasmin activity.

This mini-review aims to provide a current overview about the fast-growing group of streptococcal proteins mediating plasminogen binding.

## Streptococcal plaminogen binding proteins

**Figure 1:**
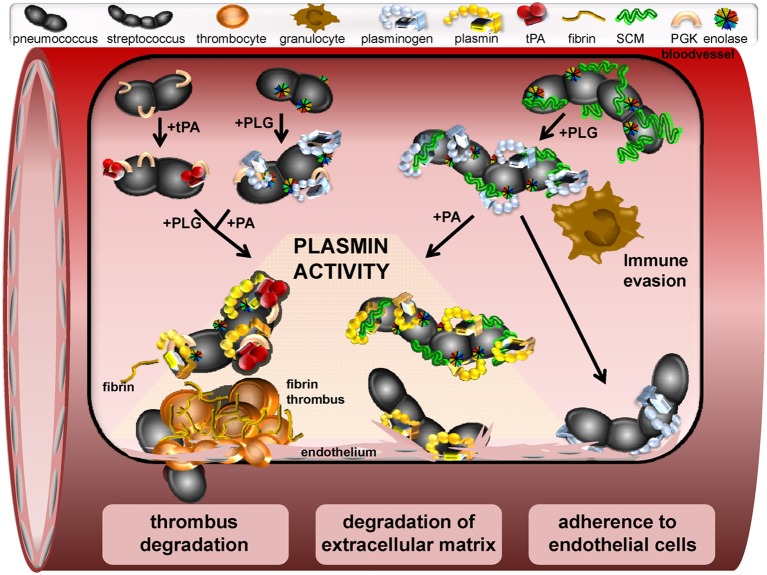
**Schematic model of a window section through a microvessel, presenting different functional mechanisms induced by interaction of streptococci with plasminogen and plasmin.** In the upper left site, binding of tPA to the pneumococcal PGK and binding of PLG to surface displayed moonlighting proteins is shown. Subsequent recruitment of PLG and a plasminogen activator leads to generation of plasmin activity on the bacterial surface. Similarly, PLG binding to streptococcal M-and M-like-proteins is followed by activation to plasmin via host-derived activators (upper right). Plasminogen binding also protects against phagocytic killing by granulocytes and macrophages and promotes adherence to endothelial cells (bottom right). Dissolution of fibrin thrombi by surface-bound plasmin-activity facilitating streptococcal transmigration is depicted on the lower left. Proteolytic degradation of extracellular matrix by recruited plasmin activity promotes bacterial-cell contact and pericellular transmigration as illustrated at the bottom center.

The relevance of plasminogen binding in streptococcal pathogenesis is documented by an increasing amount of literature published in the last years (Bergmann et al., 2001, 2005; Sun et al., 2004; Sanderson-Smith et al., 2008; Fulde et al., 2011; Siemens et al., 2011; Teles et al., 2012; Agarwal et al., 2013). The structure of bacterial receptors, their surface linkage, and their mechanism of plasminogen binding differ significantly. Based on this, three groups of proteins can be assigned (Table 1): (a) the M- and M-like proteins of pyogenic streptococci, e.g., *S. canis*, GAS, and *S. dysgalactiae* subs. *equisimilis* (*S. equisimilis*) represent a first group (Berge and Sjöbring, 1993; Sanderson-Smith et al., 2006a,b; Bergmann et al., 2011; Fulde et al., 2011, 2013a). These proteins harbor a fibrillar structure with the tendency to dimerize and are covalently linked to the bacterial cell wall via a typical LPxTG motif (Fischetti, 1991). In 1995; Wistedt and co-workers identified K2 as the interaction site of the GAS M Protein PAM (Table 1) (Wistedt et al., 1995). In contrast, SCM, a M-like protein of *S. canis*, and the M protein GCS3 of *S. equisimilis* interact with mPLG (Table 1) (Bergmann et al., 2011; Fulde et al., 2011, 2013a). (b) The second group includes several enzymes of the glycolytic pathway like enolase, phosphoglycerate kinase (PGK), and glyceraldhehyde-3-phosphate-dehydrogenase (GAPDH), a surface dehydrogenase (SDH). These proteins are transported to the bacterial surface by a yet unknown mechanism and comprise moonlighting functions. In contrast to SCM and GCS3, the plasminogen binding site for streptococcal enolase and PGK was narrowed down to the angiostatin domain (Fulde et al., 2013a,b). It is supposed that this interaction leads to an opening of the plasminogen molecule conformation and to an enhanced plasmin generation (Miles and Plow, 1985; Plow et al., 1986). The expression of a subset of plasminogen binding proteins might also increase the amount of plasminogen molecules recruited to the bacterial surface and the generated plasmin activity. Such a cooperative interaction with plasminogen was recently described for the zoonotic pathogen *S. canis*: while the M protein SCM specifically interacts with mPLG, enolase binds to angiostatin leading to formation of a multi-protein complex on the bacterial surface (Figure 1) (Fulde et al., 2013a). (c) A third group of plasminogen binding proteins comprises covalently and non-covalently cell wall linked surface proteins, e.g., the pneumococcal adherence and virulence factor B (PavB) and the pneumococcal choline esterase (Pce) (Attali et al., 2008a,b; Jensch et al., 2010). These proteins share a stalk-domain containing repetitive amino acid sequences, which mediate plasminogen and fibronectin binding and attachment to phosphoryl moieties of the cell wall.

**Table 1:**
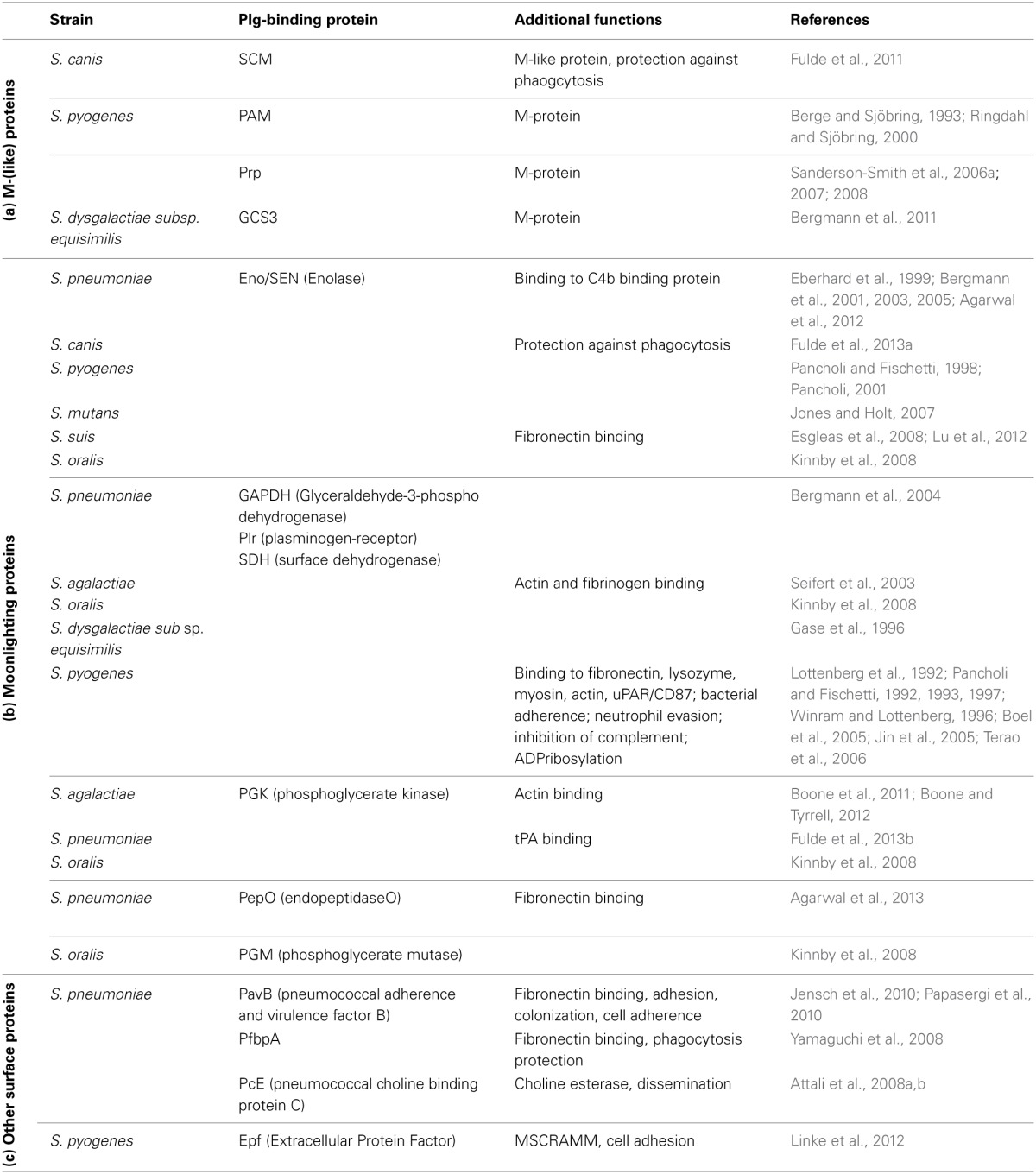
**Plasminogen-binding proteins of different streptococci and additional functions**.

## Plasminogen binding sites

For a long period of time, C-terminally located lysine residues of bacterial proteins were assumed to constitute the exclusive binding sites significantly participating in the interaction of plasminogen with host proteins like fibrin(ogen), alpha2-antiplasmin, eukaryotic enolase, and cell-surface receptors (Miles et al., 1991; Ponting et al., 1992). Meanwhile, additional internal positively charged amino acid motifs had been identified as important binding sites for the pneumococcal enolase and pneumococcal endopeptidase O (PepO) (Table 1), as well as for the M-proteins of *S. canis* and GAS (Wistedt et al., 1995; Bergmann et al., 2003; Sanderson-Smith et al., 2006b, 2007, 2012; Fu et al., 2008; Fulde et al., 2011; Agarwal et al., 2013). The differences in the localization of plasminogen binding sites can be explained by the composition of the three dimensional protein structures referring to enolase as a typical example. In contrast to the dimeric eukaryotic enolase, streptococcal enolase is usually present in an octameric form (Ehinger et al., 2004; Lu et al., 2012). The complex globular structure of the octameric molecule hides C-terminal lysine residues in interdimer grooves, thereby maintaining structural integrity. Similarly, an internal binding site was recently discovered for the dimeric PGK of *S. agalactiae* and of *S. pneumoniae*, and also for the M-protein PAM of *S. pyogenes* (Boone and Tyrrell, 2012; Fulde et al., 2013b). The interaction of the M-like protein PAM with plasminogen is mediated by two N-terminally located repeat sequences designated a1 and a2. Interestingly, in contrast to what is known for other binding motifs, an arginine, and histidine, but not lysine represent the essential amino acids mediating protein-protein interaction (Walker et al., 2005; Sanderson-Smith et al., 2007). In conclusion, despite the variations in structure and amino acid composition of plasminogen binding motifs, the presence of positively charged amino acids in a hydrophobic surrounding seems to constitute the principal requirement for binding to plasminogen.

## Plasminogen-mediated interaction

Plasminogen circulates as a mono-chained multi-domain protein in the blood of vertebrates (Pollanen et al., 1991). Even without activation, recruitment of plasminogen to the bacterial surface has been reported as pivotal pathogenicity mechanism promoting bacterial attachment to cell surfaces (Lottenberg et al., 1994). Remarkably, intranasal mouse infection studies demonstrated that plasminogen recruitment to pneumococci significantly contribute to virulence in mice (Bergmann et al., 2003). Bacterial interaction with the host fibrinolytic system represents a double edged sword. While plasminogen binding to some commensals enhances bacterial colonization and protects tissues against access by pathogens, subversion of plasmin-mediated proteolysis by pathogens promotes infection with occasionally severe pathophysiological consequences. Several publications elucidate detailed mechanisms of plasmin activity recruited on streptococcal surface via single streptococcal plasminogen binding proteins (refer to references listed in Table 1).

## Plasminogen binding proteins promote attachment to proteins of the extracellular matrix

Interaction of streptococcal enolase with plasminogen has been reported for many species of the genus *Streptococcus* (Pancholi and Fischetti, 1992; Bergmann et al., 2001; Itzek et al., 2010; Fulde et al., 2013a). Its role in pathogenicity has been analyzed using a variety of *in vivo* and *ex vivo* infection models (Pancholi and Fischetti, 1998; Pancholi and Chhatwal, 2003; Bergmann et al., 2005, 2013; Agarwal et al., 2012). In addition, many moonlighting proteins have been characterized as adhesive molecules mediating streptococcal binding to proteins of the extracellular matrix (ECM) like fibronectin (Table 1) (Pancholi and Fischetti, 1992; Seifert et al., 2003; Bergmann and Hammerschmidt, 2006; Esgleas et al., 2008; Paterson and Orihuela, 2010; Bernardo-Garcia et al., 2011; Voss et al., 2012). This interaction promotes the contact of the bacteria with epithelial and endothelial cell surfaces (Esgleas et al., 2008) and contributes to bacterial colonization of host niches. Importantly, the cooperative binding activity of plasminogen and fibronectin is not only restricted to surface-displayed glycolytic enzymes but has also been demonstrated for non-glycolytic proteins, i.e., PavB, PfbA/B, and PepO of *S. pneumoniae* and Epf of GAS (Table 1) (Yamaguchi et al., 2008; Jensch et al., 2010; Papasergi et al., 2010; Linke et al., 2012; Agarwal et al., 2013).

## Role of plasminogen in bacterial-host cell adhesion and internalization

In addition to the support of bacterial colonization, binding of ECM-proteins to bacteria has been shown to promote activation of fibronectin-specific integrin receptors, which induces bacterial uptake (Hoffmann et al., 2010; Jensch et al., 2010). Similar to fibronectin, plasminogen mediates adherence of GAS to integrin receptors and triggers bacterial internalization into the cells (Siemens et al., 2011). Interestingly, recent data also demonstrates an adhesive effect of plasminogen when already bound to cell surfaces of epithelial and endothelial cells (Figure 1). As an example, surface-exposed enolase was identified as mediator for plasminogen-dependent bacterial attachment (Table 1) (Bergmann et al., 2013). In contrast to what has been shown for internalization of GAS, pneumococcal uptake in epithelial cells was not promoted by non-activated plasminogen. This strongly indicates an involvement of additional, yet unknown co-factors in cell entry mechanisms of GAS (Siemens et al., 2011).

Considering the conformation of plasminogen, two further interesting observations with respect to cell adhesion have been reported: while coating of pneumococci with Glu-plasminogen decreases attachment, pre-incubation of M-protein expressing *S. equisimilis* with N-terminally truncated Lys-plasminogen resulted in enhanced bacterial adherence to nasopharyngeal cells (Bergmann et al., 2011). Since Lys-plasminogen possesses an open molecule form, the adhesive capacity may reflect a conformation dependent effect, as already mentioned in the literature (Marshall et al., 1994; Lahteenmaki et al., 2005).

## Plasmin-mediated interaction

Streptococci differ not only in their repertoire of plasminogen binding proteins but also in their intrinsic ability to activate the zymogen. While GAS and *S. equisimilis* express streptokinases, *S. canis*, *S. pneumoniae*, and most of the oral streptococci require host-derived uPA or tPA to convert plasminogen in its active form plasmin (Bergmann et al., 2005; Bergmann and Hammerschmidt, 2007; Kinnby et al., 2008; Itzek et al., 2010; Fulde et al., 2011). Although direct binding of plasminogen activators to streptococcal surface is not required for plasmin activation, a recent publication demonstrates a direct binding of tPA to the pneumococcal moonlighting protein PGK, which has been shown to promote plasminogen activation (Fulde et al., 2013b). In contrast to the cleavage mechanism by uPA and tPA, streptokinases form a protein complex which activates the enzyme domain of plasminogen (Boxrud and Bock, 2000; Boxrud et al., 2004). Moreover, immobilization of plasmin in fibrin fibers or on surfaces of cells protects the enzyme against inhibition by its major inhibitor the serpin α2 antiplasmin and also against inactivation via several streptokinase variants (Plow et al., 1986; Hall et al., 1991; Cook et al., 2012). The capture of plasminogen to the bacterial surface via non-activating plasminogen binding proteins induces conformational changes, which promote generation of plasmin activity by host-activators, although no protective effect against α2 antiplasmin inhibition is gained (Miles and Plow, 1985; Plow et al., 1986). Nevertheless, conversion of plasminogen to plasmin equips the bacteria with efficient proteolytic activity, which is targeted against plasmin substrates of the host.

## Degradation of fibrin thrombi and components of the extracellular matrix

Severe systemic streptococcal infections are frequently accompanied by enhanced vascular coagulation, which entraps bacteria in fibrinous thrombi (Abraham, 2000; Gunther et al., 2000). Hence, utilization of proteolytic activity has been shown to promote bacterial escape from these entrapments and enable transmigration through ECM and vascular barriers (Lahteenmaki et al., 2001). In this regard, incubation of semi-synthetic fibrin thrombi with plasmin-coated pneumococci or *S. canis* resulted in a complete dissolution of the fibrin bundles (Figure 1) (Bergmann et al., 2005; Fulde et al., 2011). Even in the absence of a streptokinase, plasminogen recruitment is accomplished via a subset of surface displayed plasminogen binding proteins acting in a compensatory manner. Fibrinogen serves as a major target for plasmin proteolysis and has been identified as an important cofactor in streptokinase-dependent plasmin activation. The reported fibrinogen binding of group A and B streptococci has also been shown to improve the efficiency of fibrin clot degradation by plasmin independent of streptokinase activity (Seifert et al., 2003; Olsen et al., 2009).

In addition to fibrin degradation, recruitment of plasmin results in enhanced degradation of ECM glycoproteins like fibronectin and laminin, which weakens the matrix integrity and might provide a benefit for dissemination of non-motile streptococci (Figure 1) (Liotta et al., 1981; Bergmann et al., 2005; Attali et al., 2008a; Fulde et al., 2013a). Moreover, plasmin-mediated cleavage of cellular junction proteins is supposed to promote subsequent pericellular transmigration of bacteria, as has been proposed for group A streptococci by Pancholi and colleagues and for pneumococci by Attali and colleagues (Pancholi et al., 2003; Attali et al., 2008b). The entirety of reported functional effects of recruited plasmin activity elucidates the mechanisms of plasminogen recruitment as a key strategy of streptococci to facilitate their transmigration through tissue barriers thereby establishing an infection.

## The role of plasminogen and plasmin in streptococcal immune evasion

Recruitment of host proteins to the streptococcal surface has been described as an efficient mechanism to circumvent innate immune strategies (Valenti-Weigand et al., 1996; Courtney et al., 2006; Yamaguchi et al., 2008). Recent reports also demonstrate the protective function of streptococcal plasminogen thereby neutralizing the host immune defense. For example, plasminogen-binding to GAS markedly reduced phagocytic killing by macrophages (Figure 1, Siemens et al., 2011). Furthermore, disruption of plasminogen binding capacity significantly reduced virulence of GAS in a murine model of invasive streptococcosis (Sanderson-Smith et al., 2008). Here, the mortality of transgenic mice expressing human plasminogen was markedly increased after infection with GAS (Sun et al., 2004; Sanderson-Smith et al., 2008). This was attributed to streptokinase expression and confirmed the high impact of plasmin generation for severe infections in humans (Sun et al., 2004). Moreover, inactivation of genes encoding either PAM or streptokinase attenuated the pathogenic potential of many GAS strains isolated from skin infections (Kalia and Bessen, 2004). A similar phenotype was recently reported for the zoonotic pathogen *S. canis*: the cooperative binding of SCM and surface-exposed enolase to plasminogen leads synergistically to an increase in the anti-phagocytic capacity as compared to the plasminogen binding activity of each of them (Figure 1) (Fulde et al., 2013a).

Plasminogen binding proteins also shield bacteria from the attack of the complement system. The GAPDH of GAS binds and inhibits the chemotactic function of the complement factor C5a (Boel et al., 2005; Terao et al., 2006). Immune-modulating activities have also been reported for the pneumococcal plasminogen binding proteins enolase and PepO. Recruitment of C4b-binding protein to the pneumococcal surface via enolase leads to a decrease in C3b deposition and represents an effective mechanism of human complement control (Agarwal et al., 2012). Furthermore, immobilization of proteolytic plasmin activity on the pneumococcal surface via PepO leads to C3b cleavage and weakens the complement-based immune defense (Agarwal et al., 2013). Another protection mechanism is directed against the activity of antimicrobial peptides such as the cathelicidin LL-37, which is cleaved by streptococcal surface-bound plasmin (Figure 1) (Hollands et al., 2012). Thus, streptococcal interaction with the plasminogen system provides powerful protective strategies against various branches of immune defense.

## Concluding remarks

The increasing amount of publications on bacterial-plasminogen interaction reflects the high relevance of this topic in infection biology. The genus *Streptococcus* comprises around 60 species exhibiting commensal as well as pathogenic properties. Especially the pathogenic species like GAS, *S. pneumoniae*, *S. canis*, and *S. suis*, benefit from plasminogen recruitment in the infection process. Pyogenic streptococci like GAS, *S. equisimilis*, and *S. canis* use M and M-like proteins to interact with plasminogen/plasmin. Since M proteins constitute the primary, covalently linked proteinous structure on the surface of many streptococci, this interaction is usually strong and correlates with the pathogenic potential of the respective isolate. Furthermore, GAS and *S. equisimilis* express plasminogen activators, which convert the surface-bound zymogen to its active, proteolytic form plasmin. In contrast, streptococci lacking M-proteins and streptokinases like *S. pneumoniae* and *S. suis* primarily use a subset of surface exposed proteins partially with moonlighting functions as plasminogen receptors. These proteins associate with the bacterial surface by a yet unknown mechanism and also the mode of anchorage remains still elusive. Therefore, the interaction between these proteins and plasminogen has long been regarded as non-essential and less relevant. Nowadays, complex formation between surface-exposed glycolytic enzymes and plasminogen/plasmin constitutes a well established explanation for systemic spread of bacteria lacking M proteins or intrinsic activators such as streptokinases. A fundamental prerequisite in this context is the utilization or direct binding of host-derived plasminogen activators by plasminogen-coated bacteria to convert the surface bound zymogen into plasmin. Finally, the cooperative plasminogen binding of *S. canis* M-Protein and the moonlighting protein enolase nicely illustrates the sophisticated and efficient strategies of bacterial interaction with the fibrinolytic system of the host. Facing the problem of antibiotic resistances, a therapeutical inhibition of the interplay between streptococci and plasminogen might constitute an alternative approach to significantly reduce bacterial cell adherence, immune evasion, and tissue degradation and would provide a promising strategy for combating severe streptococcal infections.

### Conflict of interest statement

The authors declare that the research was conducted in the absence of any commercial or financial relationships that could be construed as a potential conflict of interest.
